# Estimated Costs of Sporadic Gastrointestinal Illness Associated with Surface Water Recreation: A Combined Analysis of Data from NEEAR and CHEERS Studies

**DOI:** 10.1289/EHP130

**Published:** 2016-07-26

**Authors:** Stephanie DeFlorio-Barker, Timothy J. Wade, Rachael M. Jones, Lee S. Friedman, Coady Wing, Samuel Dorevitch

**Affiliations:** 1 Division of Environmental and Occupational Health Sciences, School of Public Health, University of Illinois, Chicago, Illinois, USA; 2 National Health and Environmental Effects Research Laboratory, Office of Research and Development, U.S. Environmental Protection Agency, Research Triangle Park, North Carolina, USA; 3 School of Public and Environmental Affairs, Indiana University, Bloomington, Indiana, USA; 4 Institute for Environmental Science and Policy, University of Illinois, Chicago, Illinois, USA

## Abstract

**Background:**

The burden of illness can be described by addressing both incidence and illness severity attributable to water recreation. Monetized as cost, attributable disease burden estimates can be useful for environmental management decisions.

**Objectives:**

We characterize the disease burden attributable to water recreation using data from two cohort studies using a cost of illness (COI) approach and estimate the largest drivers of the disease burden of water recreation.

**Methods:**

Data from the NEEAR study, which evaluated swimming and wading in marine and freshwater beaches in six U.S. states, and CHEERS, which evaluated illness after incidental-contact recreation (boating, canoeing, fishing, kayaking, and rowing) on waterways in the Chicago area, were used to estimate the cost per case of gastrointestinal illness and costs attributable to water recreation. Data on health care and medication utilization and missed days of work or leisure were collected and combined with cost data to construct measures of COI.

**Results:**

Depending on different assumptions, the cost of gastrointestinal symptoms attributable to water recreation are estimated to be $1,220 for incidental-contact recreation (range $338–$1,681) and $1,676 for swimming/wading (range $425–2,743) per 1,000 recreators. Lost productivity is a major driver of the estimated COI, accounting for up to 90% of total costs.

**Conclusions:**

Our estimates suggest gastrointestinal illness attributed to surface water recreation at urban waterways, lakes, and coastal marine beaches is responsible for costs that should be accounted for when considering the monetary impact of efforts to improve water quality. The COI provides more information than the frequency of illness, as it takes into account disease incidence, health care utilization, and lost productivity. Use of monetized disease severity information should be included in future studies of water quality and health.

**Citation:**

DeFlorio-Barker S, Wade TJ, Jones RM, Friedman LS, Wing C, Dorevitch S. 2017. Estimated costs of sporadic gastrointestinal illness associated with surface water recreation: a combined analysis of data from NEEAR and CHEERS Studies. Environ Health Perspect 125:215–222; http://dx.doi.org/10.1289/EHP130

## Introduction

Epidemiological studies indicate a relationship between surface water recreation—swimming, fishing, or boating—and the occurrence of gastrointestinal (GI) illness (Cabelli 1983; Dorevitch et al. 2012b; Dufour 1984; Stevenson 1953; Wade et al. 2008). Cases of GI illness following surface water recreation are generally mild and self-limited, but in some cases result in physician office and/or emergency department (ED) visits (Dorevitch et al. 2012b; Fleisher et al. 1998).

The severity of illness attributable to water recreation may vary across demographic characteristics (Lund and O’Brien 2011) and recreation settings due to differences in host susceptibility, exposure intensity, or levels of contamination (Glynn and Bradley 1992). As a result, evaluating only frequency of GI illness attributed to water recreation may provide an incomplete accounting of health consequences of surface water recreation, and of possible benefits of efforts to mitigate those consequences. The concept of “burden of disease” incorporates both occurrence and severity of illness and offers a useful way to compare the impact of diseases across locations, exposures, and sensitive subgroups (Murray and Lopez 1996; Rice et al. 2006). Using information regarding the severity of symptoms can aid in understanding the effects of exposure to surface water, and prioritize locations for mitigation efforts such as improving stormwater or wastewater management.

To illustrate the limitations of a frequency of illness approach, consider the following example. Beaches A and B both have similar rates of GI illness. However, the GI illnesses at A frequently result in contact with a health care provider and some recreators are hospitalized, while the illnesses at B rarely result in contact with a provider. The frequency of illness alone would not identify the additional health burden at A, whereas a metric incorporating severity would.

The burden of illness concept can be challenging to characterize because it requires combining multiple elements of disease severity, including medication use, health care provider visits, hospitalizations, and missed days of work. One way to describe these elements of disease burden is to attach a monetary value to each and calculate the total cost of each illness event. This cost of illness (COI) approach has been used to evaluate the burden associated with waterborne illness (Ailes et al. 2013; Corso et al. 2003; Dwight et al. 2005). The U.S. Environmental Protection Agency (EPA) prefers the COI method because it offers a transparent and practical input to decisions about environmental policy (U.S. EPA 2007). The COI method is a tabulation of direct costs, such as medications, visits with a health care provider, ED visits, and hospitalizations; and indirect costs, such as lost productivity from missed days of work or leisure. Returning to the example, consider that illnesses at Beach A result in an annual COI estimate of $100,000 and the illnesses at Beach B result in an annual COI estimate of $10,000. The public health burden is very different at the two beaches despite the similarity in the number of illnesses. Estimating the COI among water recreators may help inform decisions about environmental management. Targeted interventions may lead to less frequency of illness and/or less severe illnesses, thus leading to reduced economic burden.

Health care utilization data were available from two cohort studies of water recreation at urban waterways, lakes, and coastal marine beaches impacted by fecal pollution. The National Epidemiological and Environmental Assessment of Recreational Water (NEEAR) study evaluated recreators swimming and wading at freshwater and marine beaches, while the Chicago Health Environmental Exposure and Recreation Study (CHEERS) evaluated incidental-contact recreation—boating, canoeing, fishing, kayaking, and rowing on waters in the Chicago, Illinois area. The overall aim of this study was to characterize the economic burden of GI symptoms attributable to water recreation among swimmers/waders, and incidental-contact water recreators within the context of these studies. A secondary goal of this study was to rank the individual components that contribute to the COI of GI illness within these two studies. Since the incidence of illness following swimming appears to differ across marine and freshwater locations (Collier et al. 2015; Zmirou et al. 2003) costs attributable to swimming and wading were calculated at all locations and at marine and freshwater locations separately.

## Methods

### Study Populations

This analysis uses data from two earlier studies of water recreation: the National Epidemiological and Environmental Assessment of Recreational Water (NEEAR), and the Chicago Health Environmental Exposure and Recreation Study (CHEERS).

NEEAR evaluated health risks (GI, respiratory, ear, eye, or skin symptoms) associated with swimming and wading, at marine and freshwater beaches potentially impacted by human fecal pollution during the summer months of 2003–2007. Beachgoers with and without beach water contact were recruited in Alabama, Indiana, Michigan, Mississippi, Ohio, and Rhode Island. A family member responded via telephone for all household members 10–12 days following recreation regarding the occurrence of symptoms following the beach visit. Recruiting, survey administration, and corresponding water sampling methods have been described previously (Wade et al. 2008, 2010). All participants provided informed verbal consent and Institutional Review Board (IRB) approval was obtained from the University of North Carolina-Chapel Hill and the Centers for Disease Control and Prevention.

CHEERS evaluated health risks of incidental-contact water recreation, including boating, canoeing, fishing, kayaking, and rowing, during the summer months of 2007–2009. Study participants were recruited from 39 locations in the Chicago, Illinois, area. A reference group of non-water recreators participated in activities such as cycling or running. The study sites were diverse and included Lake Michigan, small inland lakes, local rivers, and the Chicago River system. At the time of the study, the majority of the flow in the Chicago River system was secondary treated, but not disinfected, wastewater effluent (Rijal et al. 2011). Telephone follow-up with individual study participants occurred approximately 2, 5, and 21 days following recreation. Study protocols used in CHEERS were similar to NEEAR. Recruiting, survey administration, and water quality assessment methods for CHEERS have been described previously (Dorevitch et al. 2012b, 2015). Participants (or parent/guardian if < 18 years of age) provided written documentation of informed consent following a protocol approved by the IRB of the University of Illinois at Chicago.

Both studies collected data on health care utilization, including prescription and over-the-counter (OTC) medication use, contact (in-person or by phone) with a health care provider, visits to an ED, and hospitalizations. In addition, both studies collected information on missed days of work, school, or leisure.

### Illness Outcome Definitions

Acute gastrointestinal illness (AGI) has been defined in recent studies of recreational water exposure (Arnold et al. 2013; Dorevitch et al. 2012b, 2015; Heaney et al. 2012; Wade et al. 2006, 2008, 2010) as diarrhea (three or more loose stools in 24 hr), or vomiting, or nausea with stomachache, or nausea that interferes with daily activities, or stomachache that interferes with daily activities. Studies suggest the strongest association between gastrointestinal illness and water recreation is within 0–3 days after recreation (Arnold et al. 2013; Colford et al. 2012; Dorevitch et al. 2012b). In the current analysis, participants developing symptoms more than 3 days following participation in the field study were not considered. All participants with GI symptoms at baseline were excluded from the analysis.

### Components of Cost of Illness

We estimated the cost of illness (COI) for each study participant who developed AGI using individual-level information from both studies on health care and medication utilization and time away from work or leisure due to illness, plus additional information from other data sources (Table 1) as described in detail below. Cost data specific to the areas where participants lived was used whenever possible. We estimated the average cost of illness (COI) for each case using Equation 1:

*COI_i_* = *OTC_i_P_OTC_* + *RX_i_P_RX_* + *HCP_i_P_HCP_* + *ED_i_P_ED_* + *Hospital_i_P_HOSPITAL_* + *Productivity_i_P_PRODUCTIVITY_* [1]

**Table 1:** Sources of data.

Variable	NEEAR study	CHEERS study
Use of OTC medications (yes/no)	Did you use any OTC medications, including things like special drinks, only because of this illness?	Did you use any OTC medications, including things like special drinks, for symptoms?
Cost of OTC medications	About how much of your own household’s money was spent altogether for OTC medicines?	Costs estimated based on costs reported by NEEAR participants
Use of prescription medications (yes/no)	Did you receive a prescription for an antibiotic or other drug for illness?	Same as NEEAR
Cost of prescription medications	About how much of your own household’s money was spent altogether for these prescription medicines?	Costs estimated based on costs reported by NEEAR participants
Visits to health care providers (any or none)^*a*^	Did you consult a health care provider over the phone? Did you visit a health care provider in person?	Did you consult a health care provider on phone or in person about any symptoms? In-person visits were estimated based on the proportion of NEEAR consultations that were visits vs. phone consultations.
Cost of visits to health care provider^*b*^	Visits assumed to be coded as E/M level 3 (CPT: 99203, 99312). Costs estimated based on age-specific probability of being uninsured (DeNavas-Walt et al. 2006), probability of being a new or returning patient (Hing et al. 2010), and CPT specific costs according to ZIP code (FairHealth 2014)	Same as NEEAR
Diagnoses	What illness did the health care provider say you had?	Same as NEEAR
Visits to an emergency department^*a*^ (any or none)	Did you visit an emergency department?	Same as NEEAR
Costs of visits to emergency department^*b*^	Visits assumed to be coded as E/M level 3 (CPT: 99203, 99312). Costs estimated based on age-specific probability of being uninsured (DeNavas-Walt et al. 2006), of specific emergency department tests and procedures [2010 Illinois Hospital Discharge database (IHA 2010)], and corresponding costs according to ZIP code [FairHealth 2014; NEDS (HCUP 2016)]	Same as NEEAR
Hospital admissions^*a*^ (any or none)	Were you admitted to the hospital?	Same as NEEAR
Lost productivity (any or none)	During illness did you miss time from work? Did illness prevent you from performing daily activities such as school, recreation, vacation activities, or work around the home? Did your illness cause other household members to lose time at work?	Did symptoms prevent you from performing daily activities such as school, work, or recreation? Missed time from work based on the proportion of NEEAR participants that missed work vs. daily activities.
Days of lost productivity	If yes to each of the above: How many days? Recorded only if ≥ 1 entire day was missed	If yes: How long were you prevented from daily activities? Recorded only if ≥ 1 entire day was missed
Cost of lost productivity	Estimated using U.S. Census sex-specific per capita income (U.S. Census Bureau 2011) for the participant’s ZIP code divided by 365 days, multiplied by days of lost productivity	Same as NEEAR
Note: In NEEAR, one person answered questions for the entire household, and questions were repeated for each of five types of symptoms reported (GI, respiratory, skin, eye, and ear). In CHEERS, each participant ≥ 7 years old answered questions regarding their own symptoms, and parents answered for each child < 7 years old. Questions applied to any type of symptom, and responses were not distinguished by symptom. OTC [over-the-counter (nonprescription) medication]. ^***a***^NEEAR participants were also asked about the number of times that they consulted with a health care provider or visited an Emergency Department. In addition, they were asked about the number of days hospitalized, and whether they were given IV fluids. ^***b***^See Table S2 for specific probabilities used in the present analysis. All costs were converted to year 2007 U.S. dollars.		

where *OTC*, *Rx, HCP*, *ED*, *Hospital*, and *Productivity* indicate the occurrence of over-the-counter (OTC) or prescription (Rx) medication use, visits to a health care provider (HCP), ED or hospital, and time missed from work or leisure (productivity) for each water recreator in either study with GI symptoms. The first term (with an *i* subscript) refers to the presence or absence (1 = yes, 0 = no) of each cost element for each water recreator. The second term of each component, *P*, refers to the cost of each of those events. Three separate versions of the COI were calculated in this analysis, based on several different assumptions described below. To account for inflation, all estimated costs in U.S. dollars (USD) were converted to year 2007 (BLS 2010), the year that both NEEAR and CHEERS field studies overlapped.

It is important to note that in both studies, other symptoms (eye, ear, respiratory, and skin) were also queried in addition to GI symptoms. Symptom outcomes, used to calculate costs (OTC use, prescription medication use, HCP contact, ED visits, and missed work/leisure) were inquired about each reported symptom in NEEAR, but only asked once, in reference to all symptoms, in CHEERS.

Both studies measured OTC and prescription medication use. NEEAR also contained a measure of expenditures for medications. Participants in NEEAR were asked about the total amount of money personally spent on either OTC or prescription medications per symptom. From 2003 to 2004, participants selected from ordinal categories of expenditure amounts (see Table S1) while from 2005–2007 participants responded to the nearest whole dollar. CHEERS participants were asked about medication use, but not costs. Medication costs for CHEERS participants were therefore estimated using NEEAR medication cost data.

NEEAR participants were asked whether they visited a health care provider for their GI symptoms. We estimated a weighted average cost for each visit reported by a NEEAR participant based on several assumptions. First we assumed that the visit would be coded as a Level 3 visit (meaning that it involved a detailed history and physical exam, and medical decision-making of low complexity) Current Procedural Terminology (CPT) 99203 (for new patients) or CPT 99213 (for established patients). The cost of a Level 3 visit is higher for new patients than for established patients, because they require a more complete medical history. We assumed that 17% were new patients based on previously published estimates for visits to outpatient departments (Hing et al. 2010). In addition, we used CPT-specific costs estimated at the ZIP code–area level (first three digits) by FairHealth (2014), a non-profit organization established to collect and aggregate data on medical care costs from multiple private insurers.

CHEERS participants were asked whether they consulted a health care provider on the phone or in-person, without distinguishing between the two types of consultations. Unlike in-person visits with a provider (PMIC 2007), phone consultations are not associated with charges (West 2012). Therefore, we estimated the proportion of consultations that involved charges by assuming that 30% were by phone, based on the proportion of phone vs. in-person consultations reported by all NEEAR participants who consulted a provider about GI symptoms. All other assumptions used to derive the weighted average cost of a visit to a health care provider by a CHEERS participant corresponded to those described above for NEEAR participants.

Both studies collected self-reported data on ED visits. Each ED visit for GI illness was assigned a weighted average cost based on the following assumptions. As was the case with office visits, we assumed that the visit would be coded as a Level 3 visit corresponding to CPT 99283 and, ICD-9-CM 009 (CDC 2009) (infectious colitis, enteritis, and gastroenteritis) or 008.8 (viral gastroenteritis) (CDC 2009). Costs of these visits are determined in part by the tests and procedures performed during the visit. Therefore, we used data from the Illinois Hospital Discharge Database (IHA 2010) to estimate the probability of individual tests and procedures (see Table S2), and data from FairHealth (2014) to estimate the associated costs of each visit according to the participant’s ZIP code area, after accounting for the probability of the tests and procedures. As an alternative high-cost estimate for ED visits, we used data on the costs of ED visits for the same ICD-9-CM codes in the 2007 Nationwide Emergency Department Sample (NEDS), Health Care Cost and Utilization Project (HCUP), Agency for Healthcare Research and Quality (AHRQ) (HCUP 2016). A national estimated cost-to-charge ratio (CCR) of 0.46 was applied to convert ED charges to costs (Freidman and Owens 2007), since AHRQ does not provide CCRs for the NEDS database.

In estimating costs of visits to health care providers and costs of ED visits, we accounted for differences in costs between patients with and without health insurance by weighting the estimated cost of each visit according to the expected proportion of participants without insurance by age (DeNavas-Walt et al. 2006) (see Table S2). Low COI estimates assumed the lower bound, moderate estimates assumed the mean, and high-COI estimates assumed the upper bound of the estimated of the proportion of uninsured individuals.

Participants were asked about hospitalizations in both studies, but no water recreators were hospitalized for their symptoms. Consequently, hospitalization costs did not contribute to our estimates of the COI associated with surface water recreation.

NEEAR participants were asked whether they missed time from work during their illness, and how many days were missed. They were asked separately whether their illnesses prevented them from performing daily activities such as school, recreation, vacation activities, or work around the home, and if so, how many days of activity were missed. In contrast, CHEERS participants were asked a single question about whether they were prevented from performing daily activities such as school, work, or recreation, and if so how many total days were missed for all of these activities combined (Table 1). Therefore, to estimate the cost of lost paid work time for CHEERS participants, we assumed that the percent of the total lost time that resulted in missing paid work was the same as what was observed among NEEAR participants (20%).

In estimating the costs of lost productivity due to illness, we considered three alternative approaches. In approach 1, consistent with previous estimates of the COI associated with foodborne or other GI illnesses (Majowicz et al. 2006; Scharff et al. 2009; Scharff 2011), we estimated the cost associated only with missed days of paid work in adults. We used the average U.S. Census sex-specific per capita income for the participant’s ZIP code (U.S. Census Bureau 2011) and calculated the daily wage using annual income divided by 365 days (Dwight et al. 2005). In the last two approaches we included unpaid work and leisure time (Drummond et al. 1997; Krol et al. 2013), and days when children were sick (since sick children often require a caregiver to remain home from work) (Carabin et al. 1999; Dwight et al. 2005) as days counting toward lost productivity. In approach 2, we assumed lost leisure time or days when children are home sick would be equivalent to the daily wage estimated for paid work for an adult, and in approach 3 we calculated lost leisure time or days when children are home sick at an overtime rate (1.5 × daily wage) (Drummond et al. 1997).

To provide a range of COI estimates, we estimated costs according to low-, medium-, and high-cost scenarios. The low-cost scenario assumed the lowest values for the proportion of uninsured according to age (based on U.S. Census data) (DeNavas-Walt et al. 2006), and the lower cost estimates for ED charges (based on data from FairHealth 2014), and included costs for missed days of paid work in adults only. The medium-cost scenario used the mean estimates for the proportion uninsured and for ED charges, but counted lost leisure time in adults and sick days in children at the same wage as time lost from paid work for adults. The high-cost scenario used the highest estimate of the proportion uninsured (DeNavas-Walt et al. 2006), the higher estimates for ED charges [based on AHRQ/NEDS (HCUP 2016)] (Freidman and Owens 2007), and counted lost leisure time and children’s sick days as 1.5 times the estimated daily wage for paid work by adults (Dwight et al. 2005).

### Estimated Costs Attributable to Water Recreation

In cohort studies of water recreation, it is not possible to determine which illnesses are attributable to water recreation on an individual level. The effect of an exposure is best estimated at the population level, because symptoms in individual water recreators can be due to factors other than water exposure (foodborne illness or person-to-person spread of infectious agents). Total estimated costs attributable to water recreation were calculated as the product of the total estimated costs among water recreators and the estimated proportion of cases of illness attributable to water recreation. This calculation used data from the non-water recreators enrolled in each study. In NEEAR, the non-water recreators were beachgoers with no water contact. In CHEERS, the unexposed included joggers, cyclists, and walkers. That proportion, the attributable fraction (AF) (Rothman et al. 2008), was calculated using estimates of the predicted probability of illness in the exposed (*p*_1_) and unexposed (*p*_0_),


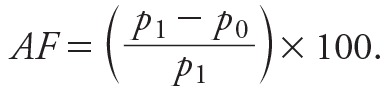
 [2]

Predicted probabilities for the exposed and unexposed recreators in each study were estimated from logistic regression models of AGI illness incidence among the exposed (water recreators) and unexposed (non-water recreators). Models assessing the occurrence of symptoms included all potential covariates, determined *a priori*, based on prior studies that have analyzed the association between water exposure and the occurrence of GI illness (Dorevitch et al. 2012b; Wade et al. 2006, 2008, 2010). Covariates included sex, race, age, the annual frequency of water recreation, washing hands prior to eating/drinking during recreation, and (for NEEAR participants only) digging in sand. Other covariates included behaviors and exposures during the follow-up period such as contact with animals; ingestion of either raw meat, raw eggs, hamburger, or fish; contact with someone with GI symptoms; and participating in water recreation during the follow-up period. Both studies collected information on the presence or absence of a pre-existing GI condition. In CHEERS, the presence or absence of diabetes, the average number of daily bowel movements at baseline, being prone to infection, or recent ingestion (within the past 7 days) of either antibiotics or antacids were controlled for in the analysis. All covariates were coded as indicated in Tables S3–S4. Model based standardization (Greenland 2004) was applied to the logistic model to estimate the probabilities of illness in the exposed (*p*_1_) and unexposed (*p*_0_). These probabilities were used to estimate the fraction of water recreators in the studies with AGI attributable to recreational water exposure (Equation 2). Bias-corrected bootstrapping (1,000 iterations) estimated confidence intervals (CI) for the attributable fraction. All analyses were conducted in Stata version 13.

Several different locations were evaluated in this analysis, therefore we also explored potential differences in total estimated costs between marine and freshwater recreators within NEEAR. Total estimated costs among marine and freshwater recreators were summed independently and multiplied by the AF to yield the total estimated costs attributable to marine and freshwater recreation within the NEEAR study.

## Results

### Study Population

In NEEAR 17,571 of 27,276 (64.4%) participants interviewed reported either swimming or wading; the remainder comprised the reference (non-water recreator) group (Table 2). In CHEERS 7,710 of 11,297 (68.2%) participants who completed telephone follow-up reported incidental-contact recreation; the remainder comprised the reference group. The demographics of the two studies were computed. The median age was higher in CHEERS and there were more children under age 10 (27.8%) in NEEAR compared to CHEERS (6.3%). The incidence of AGI among water recreators was 4.1% and 3.8% in CHEERS and NEEAR, respectively. The proportion and mean estimated costs for each component of the COI were similar across the two studies (Table 3).

**Table 2:** Characteristics of CHEERS and NEEAR water recreators.

Demographic information	CHEERS	NEEAR
Total no. (%)	7,710 (68.2)	17,571 (64.4)
Marine water (%)	NC	3,518 (20.0)
Fresh water (%)	7,710 (100)	14,053 (80.0)
Age, mean years (median)	35.4 (34.0)	25.1 (22.0)
No. < 10 years old (%)	484 (6.3)	4,757 (27.8)
No. > 65 years old (%)	233 (3.0)	184 (1.1)
Acute gastrointestinal illness (%)	315 (4.1)	675 (3.8)
Note: NC, not collected in CHEERS.		

**Table 3:** Severity characteristics of CHEERS and NEEAR water recreators with acute gastrointestinal illness.

Cost of illness component	CHEERS (*n* = 315)	NEEAR (*n *= 675)
Responses to symptoms^*a*^:
OTC medication (%)	174 (55.2)	384 (57.1)
Prescription medication (%)	15 (4.8)	77 (11.5)
Contact with health care provider (%)	40 (12.7)	112 (16.6)
Visit health care provider (%)	NC	75 (11.4)
Visit emergency department (%)	1 (0.3)	15 (2.2)
Lost work or leisure^*b*^ (%)	148 (48.0)	306 (44.1)
Mean days lost of work or leisure (median)	1.4 (1.0)	2.1 (2.0)
Missed work^*b*^ (%)	NC	63 (9.3)
Lost leisure^*b*^ (%)	NC	297 (44.1)
Any of the above response to symptoms^*a*^ (%)	225 (71.7)	487 (72.4)
Mean costs of each component of COI (2007 USD):
OTC medications	$11.34	$9.37
Prescription medications	$23.87	$32.31
Contact with health care provider^*c*^	$56.04	$44.17
Emergency department visit^*d*^	$149.94	$169.53
Emergency department visit^*e*^	$792.71	$792.71
Lost productivity	$251.17	$222.01
Note: NC, not collected in CHEERS; USD, U.S. dollars. ^***a***^Over-the-counter (OTC) medication use or prescription medication use, or contact with a health care provider, or emergency department visit, or lost time from work or leisure due to gastrointestinal symptoms. ^***b***^In CHEERS lost work/leisure inquired about in a single question, in NEEAR, separate inquires for lost time from work, and lost leisure time. ^***c***^Weighted average costs of new insured, established insured, new uninsured, and established uninsured costs. ^***d***^Costs derived from FairHealth (2014), average of insured and uninsured, and average tests performed. ^***e***^Costs derived from National Emergency Department Sample (NEDS), Health care Cost and Utilization Project (HCUP) Agency for Healthcare Research and Quality (AHRQ) (HCUP 2016), as an alternate source of data.		

### Estimated Cost of Gastrointestinal Illness

**Figure 1:**
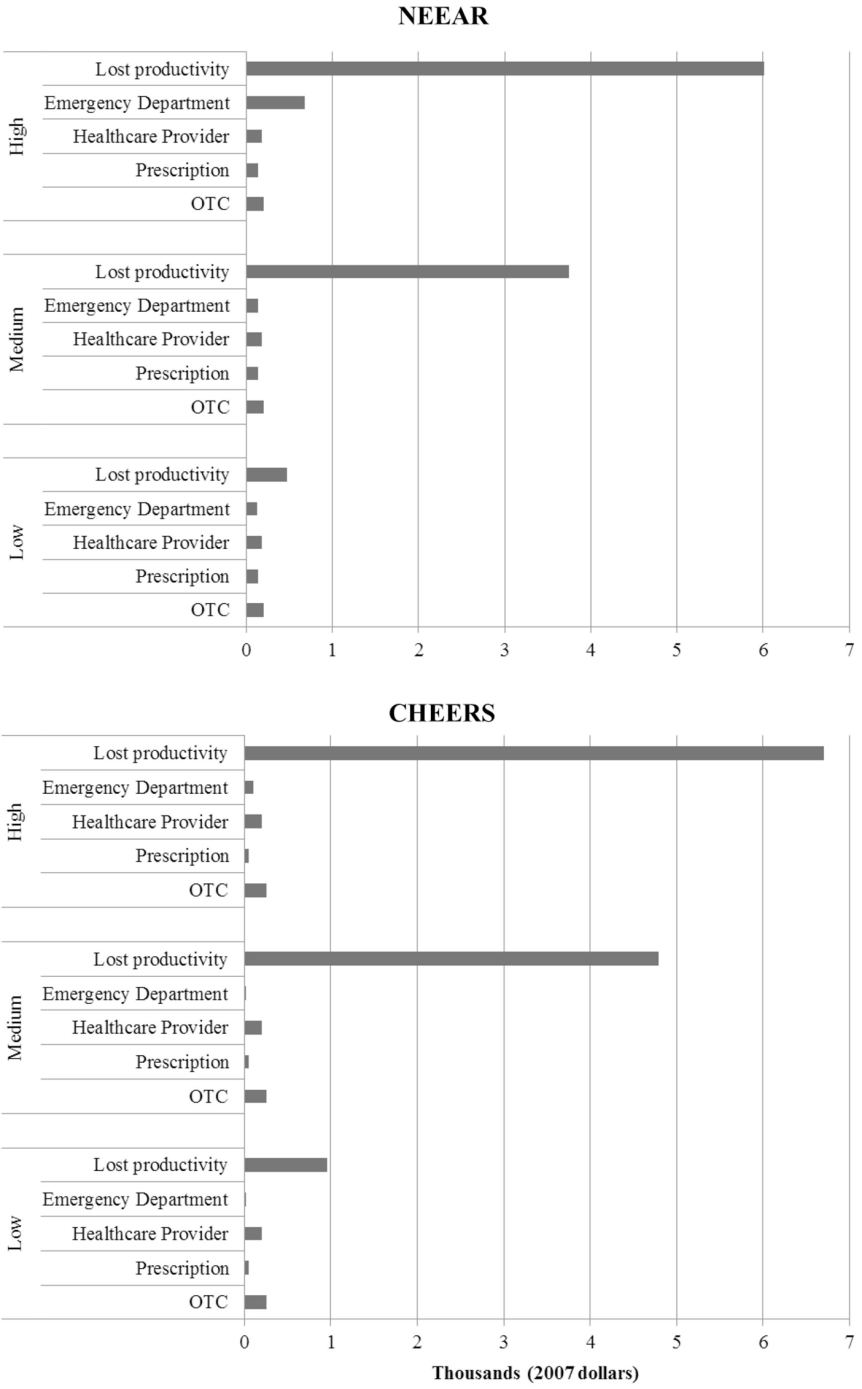
Contributions of each component of the total estimated costs of acute gastrointestinal illness per 1,000 water recreators, prior to adjusting for the attributable fraction. Low, lowest uninsured rates, lowest estimates for ED charges (FairHealth 2014), leisure time assumed not associated with cost. Medium, average uninsured rates, lowest estimates for ED charges (FairHealth 2014), leisure time assumed equivalent to daily wage. High, highest uninsured rates, highest estimates for ED charges [NEDS (HCUP 2016)], leisure time assumed equivalent to an overtime rate (1.5 × daily wage).

The low-, medium-, and high-cost assumptions resulted in a range of estimates of AGI COI (Table 4, Figure 1). In NEEAR estimated mean total COI among water recreators with AGI ranged from $46.18 to $263.10. In CHEERS, estimated mean COI among water recreators with AGI was similar to those for NEEAR, ranging from $50.31 to $250.39. Costs per water recreator ranged from $1.17 to $7.22 among NEEAR recreators, and from $1.47 to $7.31 among CHEERS recreators (Table 4). Lost productivity contributed the greatest proportion (42–90%) of the estimated total COI (Figure 1). This was true whether lost leisure time was valued as 0 × daily wage, 1 × daily wage, or 1.5 × daily wage. The second largest contributor to the estimated total COI was OTC medication use, ranging from 3% to 18% of the total costs. Note that the costs estimates summarized in Table 4 and Figure 1 do not distinguish between illness attributable to water recreation and illness among water recreators due to other causes.

**Table 4:** Estimates of the cost of acute gastrointestinal illness (2007 U.S. dollars).

Cost of illness level	Study	*n*^*a*^	Mean cost per case of AGI^*b*^	Median cost per case of AGI^*b*^	Range of cost per case of AGI^*b*^	Total costs per 1,000 water recreators^*c*^	Mean cost per water recreator^*c*^
Low: lowest uninsured rates, lowest estimates for ED charges (FairHealth), leisure time assumed to not be associated with cost	NEEAR	425	46.18	8.19	5.00–713.10	1,117.10	1.17
	CHEERS	225	50.31	28.36	3.00–580.58	1,468.13	1.47
Medium: average uninsured rates, lowest estimates for ED charges (FairHealth 2014), leisure time assumed equivalent to the daily wage	NEEAR	482	160.79	108.49	6.00–1,182.65	4,410.60	4.41
	CHEERS	225	181.71	85.54	3.00–2,865.14	5,302.69	5.30
High: highest uninsured rates, highest estimates for ED charges [NEDS (HCUP 2016)], leisure time assumed equivalent to an overtime rate (1.5 × daily wage)	NEEAR	482	263.10	162.86	6.00–2,206.79	7,217.30	7.22
	CHEERS	225	250.39	109.85	3.00–4,007.42	7,306.98	7.31
^***a***^Number of water recreators with cost data. ^***b***^Acute gastrointestinal illness. ^***c***^7,710 water recreators in CHEERS, 17,571 water recreators in NEEAR, prior to adjusting for the attributable fraction.							

### Estimated Costs Attributable to Water Recreation

The largest estimated AF [38% (95% CI: 26%, 46%)] was found among NEEAR water recreators (swimmers/waders) who developed AGI, compared to the unexposed group (Table 5). Total estimated costs attributable to water recreation per 1,000 water recreators were relatively similar between the studies overall ranging between $337.67 and $2,742.58 for participants with AGI.

**Table 5:** Probability of acute gastrointestinal illness and estimated costs (2007 U.S. dollar) attributable to water recreation, 95% CI.

Outcome measure	NEEAR	CHEERS
Probability of AGI^*a*^ if exposed (*p0*)	0.040 (0.037, 0.043)	0.044 (0.038, 0.049)
Probability of AGI^*a*^ if not exposed (*p1*)	0.025 (0.022, 0.029)	0.034 (0.027, 0.041)
Relative risk^*b*^	1.61 (1.36, 1.87)	1.30 (1.01, 1.68)
Odds ratio^*c*^	1.64 (1.39, 1.94)	1.32 (1.04, 1.68)
Attributable fraction	0.38 (0.26, 0.46)	0.23 (0.01, 0.40)
Costs attributable to water recreation per 1,000 water recreators^*d*^ ($), low-cost assumption	424.50 (290.45, 513.87)	337.67 (14.68, 587.25)
Costs attributable to water recreation per 1,000 water recreators^*d*^ ($), medium-cost assumption	1,676.03 (1,146.76, 2,028.88)	1,219.62 (53.03, 2,121.08)
Costs attributable to water recreation per 1,000 water recreators^*d*^ ($), high-cost assumption	2,742.58 (1,876.50, 3,319.96)	1,680.60 (73.07, 2,922.79)
^***a***^Acute gastrointestinal illness. ^***b***^RR = *p1*/*p0*. ^***c***^From logistic regression models (see Tables S3–S4). ^***d***^7,710 water recreators in CHEERS, 17,571 water recreators in NEEAR.		

The adjusted risk of AGI among NEEAR water recreators was comparable at marine and freshwater locations (*p* = 0.23). However, estimated costs at these locations were not. Total estimated costs per 1,000 recreators (low assumption) attributable to water recreation at marine locations [$611.61 (95% CI: $418.47–$740.36)] were significantly greater (*p* = 0.02) compared to freshwater locations [$365.94 (95% CI: $258.40–$457.16)]. Similar results were observed when comparing other assumptions (medium or high) (see Table S5). Interestingly, among working-age adults (18–44) recreating in marine water, a higher percent (37.8%) report missing work due to AGI compared to 21.3% of freshwater recreators in the same age category (*p* = 0.04).

## Discussion

This study estimated the total cost of AGI attributable to water recreation in two similar, but distinct epidemiological studies of water recreation and health. The estimated costs attributable to water recreation were similar in studies composed of swimmers and waders (NEEAR) and incidental-contact water recreators (CHEERS). Overall, estimated costs attributable to water recreation per 1,000 water recreators ranged from $337.67 to $2,742.58 for those with AGI (Table 5). Low, medium, and high assumptions were responsible for the wide range of estimated costs and in all scenarios lost productivity was the largest contributor to overall estimated costs (42–90%) (Figure 1). We also estimated that costs per 1,000 marine recreators [$611.61 (95% CI: $418.47–$740.36)] were higher compared to freshwater recreators [$365.94 (95% CI: $258.40–$457.16)]. However, other differences in socioeconomic status or location (freshwater beaches along the Great Lakes, marine beaches on the Eastern seaboard and Gulf Coast) could account for these different cost estimates.

Per-case costs of AGI estimated within these analyses are within the range of costs of similar illnesses found in the literature (see Table S6). A previous analysis (Dwight et al. 2005) estimated that the per-case cost of GI illness among surface water recreators was $42.83, yet did not include costs of medications, ED visits, or hospitalizations. Notably, Dwight et al. (2005) reported that approximately 15% of those who developed illness missed time from work or leisure. In contrast, time away from work or leisure was reported by at least 40% of water recreators with AGI in CHEERS and NEEAR (Table 3). Others have estimated that the per-case cost of mild GI illness attributable to *Cryptosporidium* in drinking water to be $166.45 (Corso et al. 2003) while the cost of community GI illness has been estimated to be $17.73 (Hellard et al. 2003). Studies on GI illness due to foodborne illness have estimated costs per case of $1,032–$1,571, depending on model assumptions (Scharff 2011). Costs associated with lost productivity are major contributors to overall COI in studies assessing costs associated with exposure to recreational water (Dwight et al. 2005), foodborne illness (Scharff et al. 2009; Scharff 2011), and studies of community gastroenteritis (Garthright et al. 1988; Hellard et al. 2003; Majowicz et al. 2006).

This analysis had several strengths. Both epidemiologic studies collected utilization of health care services and productivity data, which were incorporated into the estimated COI. Other studies evaluating COI excluded certain costs unavailable at the time of the analysis, such as medications (Dwight et al. 2005; Garthright et al. 1988), and ED visits or hospitalizations (Dwight et al. 2005). Geographically-specific cost estimates for contact with a health care provider and ED visits were obtained based on estimates from the U.S. population for insurance status and patient type. Lastly, the level of detail available for each cost estimate allowed for several versions of the COI to be calculated, resulting in a range of potential COI, rather than a single point estimate.

The current study also had several limitations. First, these two studies were not designed for evaluating economic burden, and as with many epidemiology studies, we relied upon self-reported information and cost data generated for other purposes. While illness severity information was collected from study participants, crucial cost-related information, such as insurance status, patient type (new or established), or actual costs spent on illness (other than medications) were not. Therefore, several assumptions regarding costs were necessary. It was assumed that for purposes of estimating health care provider costs, the distribution of participant insurance status (insured vs. uninsured) and patient type (new vs. established) were similar to those in national datasets. Insurance status was estimated based on age, yet age is not the only contributor to insurance status. Other factors, such as location of residence, income, and race may also influence insurance status (DeNavas-Walt et al. 2006). In addition, regional differences in medical care costs could impact our estimates since these studies were conducted in multiple states. Therefore, any comparisons between or within these two studies should be interpreted with caution.

Medication costs were estimated for CHEERS participants based on the data from NEEAR, even though the two studies were conducted in different locations. Our approach relied entirely on self-reported data about the use of medications (OTC or prescription) for GI symptoms, and did not require us to make any additional assumptions about the types of medications taken by each individual, other than assuming that costs would have been similar between the two studies. Additionally, the proportion of CHEERS participants who visited (versus phoned) a health care provider and the proportion that missed work (versus work and leisure combined) were estimated based on the observed proportions in NEEAR participants. It is unclear if these proportions are comparable across the study populations.

The calculated costs in this analysis were expected to reflect costs to the individual. Costs related to insurance claims processing, and to employers for lost productivity, among others, were not considered. The approach taken utilized the human capital method of valuing individual costs, which counts costs incurred to the sick individual, thus counts days not worked as days lost. Alternatively, the friction cost method could have been applied, which takes the “employer’s perspective” and considers costs of missed work until another employee takes over the work of the ill individual (Van den Hout 2010). Additionally, a more complete and mid-range estimate of costs would have also incorporated costs estimated to be associated with unpaid work, including caregiving or volunteering, which is becoming more prominent in economic analyses (Krol et al. 2013).

Another consideration is that a portion of CHEERS and NEEAR sites were impacted by discharges from wastewater treatment plants. Certain sites within CHEERS were known to contain high concentrations of fecal indicator organisms (Dorevitch et al. 2015). The current analysis assesses costs within the context of these two studies of water recreation in effluent-dominated inland and coastal waters. Therefore, these estimates may not be generalizable to all types of water recreation, especially recreation in uncontaminated waters where illness occurrence may be lower and less severe. In addition, these results may also not be generalizable in locations with different climactic patterns, in areas where the pathogen profile may result in differing illness severity, or in areas with emerging drug-resistant pathogens.

The main focus of this analysis was to estimate costs among those who develop symptoms within 3 days of recreation based on findings of prior studies (Arnold et al. 2013; Colford et al. 2012; Dorevitch et al. 2012b). The case definitions used exclude those who develop symptoms beyond this time-window. Therefore, our results may ultimately underestimate total costs, since other illnesses potentially attributable to water recreation with longer incubation periods were not included.

Additionally, we chose to assess ED and hospitalization costs according to ICD-9-CM codes (CDC 2009) for unidentified gastrointestinal illnesses. It is not well understood which particular infectious agents are directly responsible for excess cases of gastrointestinal illness among water recreators in epidemiology studies, since specific pathogens responsible for waterborne illnesses have not been identified in stool samples of symptomatic study participants (Dorevitch et al. 2012a; Jones et al. 1991). However, if we included more specific outcomes, we would expect these costs to vary considerably.

In addition, we assumed that, at the individual level, the utilization of health care services (such as tests and treatments in the ED) were similar for people with GI illness due to water recreation and people with GI illness due to other causes. While we are unable to validate this assumption [at the level of individuals, the cause(s) of illness are not known], however COI of GI illness in other contexts are within the same range of our estimates (see Table S6).

Lastly, a weighted average, taking into account variations such as insurance status and patient type, was used to estimate individual cost components, which did not directly incorporate variability of the cost estimates. This lack of variability in a single estimate was to some degree mitigated by incorporating several different sets of assumptions to create a range of total costs of GI illness.

## Conclusion

We estimate that the cost of illness attributable to water recreation (in year 2007 USD) was $1,220 (range $338–1,681) for every 1,000 people who engaged in canoeing, kayaking, fishing, rowing, or paddling in the Chicago area (in the CHEERS study); and $1,676 (range $425–2,743) for every 1,000 people engaged in swimming or wading at freshwater and marine locations in six states (in the NEEAR study).The costs per 1,000 water recreators could be reduced if interventions designed to reduce fecal indicator organisms in the water lead to lower incidence and/or less severe symptoms. The burden of recreational waterborne illness can be reduced by prioritizing beaches with high burden for intervention, where high burden reflects heavy beach use, frequent illness and/or severe illness. The cost of illness information described here should be useful in putting into context costs of beach monitoring and notification programs, costs of upgrading stormwater and wastewater infrastructure, and other interventions that may help reduce the burden of illness due to surface water recreation.

**Editor’s Note:** Corrections were made to the NEEAR data in Table 5 to reflect a minor adjustment to the calculations. These data were corrected in the text before advance publication, so only the values in Table 5 have changed. The authors regret the error.

## Supplemental Material

(171 KB) PDF
