# Supplementary material for: Estimated Costs of Sporadic Gastrointestinal Illness Associated with Surface Water Recreation: A Combined Analysis of Data from NEEAR and CHEERS Studies
Source: Environ Health Perspect. 2016 Jul 26;125(2):215–22. doi: 10.1289/EHP130 (PMC5289902; doi:10.1289/EHP130)
Supplement: (171 KB) PDF [file EHP130.s001.acco.pdf]

Environ Health Perspect
DOI: 10.1289/EHP130

Note to readers with disabilities: *EHP* strives to ensure that all journal content is accessible to all readers. However, some figures and Supplemental Material published in *EHP* articles may not conform to 508 standards due to the complexity of the information being presented. If you need assistance accessing journal content, please contact ehp508@niehs.nih.gov. Our staff will work with you to assess and meet your accessibility needs within 3 working days.

Supplemental Material

Estimated Costs of Sporadic Gastrointestinal Illness Associated with Surface Water Recreation: A Combined Analysis of Data from NEEAR and CHEERS Studies

Stephanie DeFlorio-Barker, Timothy J. Wade, Rachael M. Jones, Lee S. Friedman, Coady Wing, and Samuel Dorevitch

Table of Contents

Table S1: Categorical responses to the amount of money spent on over-the-counter (OTC) or prescription medications for NEEAR participants from 2003–2004

Table S2: Estimated patient information to be used to estimate cost of illness

Table S3: Full model for the relationship between Acute Gastrointestinal Illness (AGI) and water contact, among participants in the NEEAR study

Table S4: Full model for the relationship between Acute Gastrointestinal Illness (AGI) and water contact, among participants in the CHEERS Study

Table S5: Costs attributable to water recreation (2007 USD), marine versus freshwater recreators in NEEAR

Table S6: Reported mean costs per case of gastrointestinal and other related illnesses (2007 USD)

References

Table S1: Categorical responses to the amount of money spent on over-the-counter (OTC) or prescription medications for NEEAR participants with gastrointestinal symptoms from 2003–2004

Categories of OTC Costs ($)	Midpoint OTC cost ($) (imputed value)	Categories of Prescription Costs ($)	Midpoint prescription cost ($) (imputed value)
0–10.00	5.00	0–25.00	12.5
11.00–25.00	18.00	26.00–50.00	38.00
26.00–50.00	38.00	51.00–75.00	63.00
51.00–75.00	63.00	76.00–100.00	88.00
76.00–100.00	88.00	101.00–150.00	125.50
101.00–150.00	125.50	151.00–200.00	175.50
151.00–200.00	175.50	>200.00	250.00
>200.00	250.00	--	--

Table S2: Estimated patient information to be used to estimate cost of illness

Parameter	Probability (CI)[a]
Uninsured status [b]	
(Probability being uninsured (90% CI))	
Total	0.159 (0.157, 0.161)
By age category	
under 18	0.112 (0.109, 0.115)
18–24	0.306 (0.299, 0.313)
25–34	0.264 (0.258, 0.300)
35–44	0.188 (0.183, 0.193)
45–64	0.145 (0.142, 0.148)
≥65	0.130 (0.110, 0.150)
Patient Type [c]	
New	0.171 (0.168, 0.173)
Established	0.829 (0.827, 0.832)
Emergency Department Test and Procedures [d]	
Electrolyte panel	0.189 (0.113, 0.264)
Blood Glucose	0.189 (0.113, 0.264)
Renal Function	0.189 (0.113, 0.264)
Complete Blood Count (CBC)	0.189 (0.113, 0.264)
Urinalysis	0.132 (0.070, 0.200)
Urine Pregnancy Test [e]	0.650 (0.001, 0.127)
Stool Culture	0.280 (0.000, 0.600)
Intravenous (IV) Hydration Infusion	0.104 (0.004, 0.163)

[a] 95% CIs are provided, unless otherwise specified
[b] DeNavas-Walt et al. 2006
[c] Hing et al. 2010
[d] 2010 Illinois Hospital Discharge Database (IHDD 2010), among those in emergency departments with GI symptoms (ICD-9-CM: 009, 008.8)
[e] Assessed among females 13–55

Table S3: Full model for the relationship between Acute Gastrointestinal Illness (AGI) and water contact, among participants in the NEEAR study

Covariate	Level	Odds Ratio (95% CI)	Standard Error
**Any water contact (ref=No)**	**Yes**	**1.64 (1.39, 1.94)**	**0.139**
Age (ref=20-54)	0-10	1.04 (0.87, 1.26)	0.099
	11-19	0.75 (0.61, 0.94)	0.084
	55+	0.74 (0.55, 1.00)	0.112
Sex (ref=male)	Female	1.16 (1.01, 1.34)	0.082
Race (ref=white)	Black	0.93 (0.67, 1.28)	0.151
	Asian	0.67 (0.33, 1.36)	0.243
	American Indian/Alaskan Native	0.94 (0.23, 3.85)	0.676
	Hispanic	1.08 (0.85, 1.37)	0.131
	Multi-race	0.22 (0.05, 0.89)	0.156
	Other	1.08 (0.55, 2.12)	0.373
Chronic GI condition (ref=No)	Yes	1.94 (1.40, 2.68)	0.321
Contact with someone with GI illness (ref=No)	Yes	2.02 (1.67, 2.44)	0.195
Ingest raw meat (ref=No)	Yes	1.45 (1.18, 1.78)	0.154
Ingest raw/runny eggs (ref=No)	Yes	0.91 (0.64, 1.29)	0.163
Ingest shellfish (ref=No)	Yes	0.85 (0.57, 1.25)	0.167
Contact with familiar animals (ref=No)	Yes	1.27 (1.05, 1.51)	0.117
Contact with unknown/unfamiliar animals (ref=No)	Yes	1.15 (0.94, 1.41)	0.119
Wash hands prior to eating/drinking (ref=No)	Yes	0.67 (0.36, 1.24)	0.210
Dig in Sand (ref=No)	Yes	1.28 (1.09, 1.49)	0.101
Recreation during follow-up (ref=No)	Yes	0.88 (0.76, 1.02)	0.065
Frequency of water recreation annually (ref=0-2 times)	2-4 times	1.07 (0.91, 1.26)	0.089
	5 times or more	1.18 (1.00, 1.41)	0.103
Beach (ref=Beach 1)	Beach 2	0.89 (0.62, 1.28)	0.163
	Beach 3	0.50 (0.34, 0.73)	0.096
	Beach 4	0.93 (0.67, 1.31)	0.160
	Beach 5	0.55 (0.41, 0.74)	0.084
	Beach 6	0.75 (0.53, 1.04)	0.128
	Beach 7	0.76 (0.55, 1.02)	0.122

Table S4 Full model for the relationship between Acute Gastrointestinal Illness (AGI) and water contact, among participants in the CHEERS study

Covariate	Level	Odds Ratio (95% CI)	Standard Error
**Any water contact (ref=No)**	**Yes**	**1.32 (1.04, 1.68)**	**0.160**
Age (ref=20-54)	0-10	0.54 (0.33, 0.90)	0.140
	11-19	0.86 (0.62, 1.20)	0.145
	55+	0.50 (0.35, 0.71)	0.089
Sex (ref=male)	Female	1.29 (1.06, 1.59)	0.136
Race (ref=white)	Black	1.91 (1.38, 2.64)	0.315
	Hispanic	1.35 (0.93, 1.95)	0.253
	American Indian	1.84 (0.43, 7.85)	1.362
	Hawaiian/Pacific Islander	0.71 (0.10, 5.23)	0.724
	Asian	0.84 (0.50, 1.39)	0.218
	Other Race	1.82 (1.04, 3.20)	0.524
	Mixed Race	1.70 (0.85, 3.42)	0.606
Chronic GI condition (ref=No)	Yes	1.94 (1.31, 2.86)	0.385
Average bowel movements per day		1.32 (1.14, 1.53)	0.099
Diabetes (ref=No)	Yes	1.61 (0.95, 2.72)	0.430
Prone to Infection (ref=No)	Yes	0.88 (0.47, 1.64)	0.281
Contact with someone with GI illness (ref=No)	Yes	1.32 (0.85, 2.07)	0.303
Ingest raw meat (ref=No)	Yes	1.11 (0.69, 1.79)	0.270
Ingest hamburger (ref=No)	Yes	1.16 (0.92, 1.45)	0.132
Ingest fresh produce	Yes	0.85 (0.62, 1.17)	0.138
Ingest pre-packaged sandwich	Yes	1.46 (1.01, 2.13)	0.279
Ingest raw/runny eggs (ref=No)	Yes	1.21 (0.77, 1.91)	0.283
Ingest shellfish (ref=No)	Yes	1.02 (0.68, 1.54)	0.214
Contact with dog/cat (ref=No)	Yes	0.97 (0.78, 1.20)	0.106
Contact with other animals (ref=No)	Yes	1.26 (0.88, 1.81)	0.231
Wash hands prior to eating/drinking (ref=No)	Yes	1.62 (0.69, 3.78)	0.701
Antibiotics past 7 days (ref=No)	Yes	1.06 (0.66, 1.72)	0.261
Antacid use past 7 days (ref=No)	Yes	1.24 (0.87, 1.78)	0.226
Recreation during follow-up (ref=No)	Yes	0.97 (0.77, 1.21)	0.111
Frequency of water recreation annually (ref=0-4 times)	5-10 times	1.15 (0.82, 2.07)	0.203
	11 times or more	0.87 (0.63, 1.21)	0.145

Table S5: Costs attributable to water recreation (2007 USD), marine versus freshwater recreators in NEEAR

**Cost of Illness Level**	**Acute Gastrointestinal Illness, 0-3 days: Fresh**	**Acute Gastrointestinal Illness, 0-3 days: Marine**	**Kruskal-Wallis pvalue**
Costs attributable to water recreation per 1,000 water recreators ($), Low cost assumption	365.94 (258.40-457.16)	611.61 (418.47-740.36)	0.0164
Costs attributable to water recreation per 1,000 water recreators ($), Medium cost assumption	1,510.06 (1,066.28-1,886.50)	2,145.89 (1,468.24-2,597.66)	0.0285
Costs attributable to water recreation per 1,000 water recreators ($), High cost assumption	2,449.06 (1,729.33-3,059.59)	3,601.78 (2,464.38-4,360.05)	0.0215

3,518 marine water recreators in; 14,053 fresh water recreators

Table S6: Reported mean costs per case of gastrointestinal and other related illnesses (2007 USD)

Type	Details	Original Cost	Cost in 2007 USD [a]	Reference
Acute Gastrointestinal Illness	Swimming/wading, Medium Assumptions:		$160.79	Current study, medium level costs
Acute Gastrointestinal Illness	incidental-contact, Medium Assumptions:		$181.71	Current study, medium level costs
GI, recreational		$36.58	$42.83	Dwight et al. 2005
GI, *Cryptosporidium*, drinking water	Mild illness:<br>Moderate illness:<br>Severe Illness:	$116.00<br>$475.00<br>$7,808.00	$166.45<br>$681.57<br>$11,203.64	Corso et al.2003
Community GI		A$18.08	$17.73	Hellard et al. 2003
Intestinal Infectious Disease	No physician consult:<br>Physician consult:<br>Hospitalization:	$215.00<br>$348.00<br>$3,038.00	$414.30<br>$670.59<br>$5,854.14	Garthright et al. 1988
Community GI		Can$1,089.00	$1,057.77	Majowicz et al. 2006
GI, foodborne	Basic model assumptions [b]:<br>Enhanced model [c]:	$1,068.00<br>$1,626.00	$1,032.18<br>$1,571.47	Scharff, 2011
GI, STEC [d]	GI only:<br>HUS [e]:<br>ESRD [f]:	€126.00<br>€25,713.00<br>€1,223,998.00	$179.08<br>$36,545.08<br>$1,739,629.95	Tariq et al., 2011

[a] Published costs converted to US dollars. All adjusted to 2007 USD.
[b] Lost productivity calculated using the daily wage
[c] Monetized quality adjusted life year (QALY), replaced daily wage
[d] STEC: Shiga toxin-producing *E. coli*
[e] HUS: Hemolytic uremic syndrome
[f] ESRD: End-stage renal disease

References

Corso PS, Kramer MH, Blair KA, Addiss DG, Davis JP, Haddix AC. 2003. Cost of illness in the 1993 waterborne cryptosporidium outbreak, Milwaukee, Wisconsin. Emerg infectdis 9:426-431.

DeNavas-Walt C, Lee CH, Proctor BD. 2006. Income, poverty, and health insurance coverage in the United States: 2005. (United States Bureau of the Census, Aug 2006, 86 pp U6 -U8.

Dwight RH, Fernandez LM, Baker DB, Semenza JC, Olson BH. 2005. Estimating the economic burden from illnesses associated with recreational coastal water pollution: A case study in orange county, California. J Environ Manage 76:95-103.

FairHealth. 2014. Fairhealth consumer cost lookup. Available: http://fairhealthconsumer.org/ [Accessed July 1, 2014].

Garthright WE, Archer DL, Kvenberg JE. 1988. Estimates of incidence and costs of intestinal infectious diseases in the United States. Public health reports 103:107.

Hellard ME, Sinclair MI, Harris AH, Kirk M, Fairley CK. 2003. Cost of community gastroenteritis. J Gastroenterol Hepatol 18:322-328.

Hing E, Hall MJ, Ashman JJ, Xu J. 2010. National hospital ambulatory medical care survey: 2007 outpatient department summary. National health statistics reports 28:1-32.

IHA (Illinois Hospital Association Business Solutions). 2010. COMPdata. Naperville, IL: Illinois Hospital Association.

Majowicz SE, McNab WB, Sockett P, Henson S, Dore K, Edge VL, et al. 2006. Burden and cost of gastroenteritis in a Canadian community. J Food Protect 69:651-659.

Scharff RL. 2011. Economic burden from health losses due to foodborne illness in the United States. J Food Protect 75:123-131.

Tariq L, Haagsma J, Havelaar A. 2011. Cost of illness and disease burden in the Netherlands due to infections with Shiga toxin-producing *Escherichia coli O157*. J Food Protect 74:545-552.
